# Supplementary material for: Are carbon emissions trading and green financial instruments synergistic? -Comprehensive quantitative research based on content analysis
Source: PLoS One. 2024 Mar 7;19(3):e0298601. doi: 10.1371/journal.pone.0298601 (PMC10919601; doi:10.1371/journal.pone.0298601)
Supplement: S1 Data — (ZIP) [file pone.0298601.s001.zip › Data/地级市Z_F工作报告.zip!/║ú─╧/║ú┐┌╩╨/└┤╫╘╣1⁄2╓┌║┼í╛┬φ┐╦╩2╛▌═°í┐_2016.docx]

各位代表：

现在，我代表市人民政府向大会报告工作，请连同《海口市国民经济和社会发展第十三个五年规划纲要（草案）》一并审议，并请市政协委员和其他列席人员提出意见。

一、2015年及“十二五”工作回顾

刚刚过去的2015年，是我市经济社会发展进程中很不平凡的一年。面对复杂多变的宏观形势，在省委、省政府和市委的坚强领导下，在市人大、市政协的监督与支持下，我们认真学习贯彻落实党的十八大、十八届三中、四中、五中全会和习近平总书记系列重要讲话精神，坚持稳中求进的总基调，主动适应新常态，着力稳增长、促改革、调结构、惠民生、强管理，认真践行“三严三实”，大力弘扬“四种精神”，全面开启“双创”模式，主动履职担当，苦干实干快干，实现了稳增长和抓“双创”两促进、双丰收。

预计全年地区生产总值1170亿元，增长8.5%；全社会固定资产投资1012亿元，增长23.2%；地方一般公共预算收入111.5亿元，增长10%；社会消费品零售总额595.5亿元，增长6.6%；城镇和农村常住居民人均可支配收入分别为28520元和11693元，分别增长7.5%和10%；居民消费价格指数涨幅1.2%、城镇登记失业率0.9%，远低于控制目标。市十五届人大六次会议确定的主要目标胜利完成！

一年来，主要抓了以下七个方面的工作：

（一）综合施策实现经济逆势上扬

准确把握经济运行动态，加大精准调控力度，经济逐季向好、稳中趋快。

狠抓重点项目扩大投资。67个省重点项目完成投资452亿元，占年度计划的138%，呈现出开工多、进度快、投资大的特点。新海港区一期开港，海秀快速路建设全线提速，垃圾综合处理二期稳步推进，美兰机场二期、地下综合管廊、新海港区二期、马村港区三期等开工建设。棚改全面提速，优化拆迁补偿政策，全面推行货币化安置，完成投资130亿元、改造1.2万户、惠及4万人，超额完成任务。全力以赴开展“投资项目百日大会战”，237个项目开工率98.3%，完成计划投资额的181%、占全省的一半以上。在全省“百日大会战”考核评比中，我市揽获7项大奖，取得历史性突破。其中综合特等奖、重点项目上台阶奖是全省唯一，招商签约、棚改、信息基础设施建设、项目储备和专项建设基金项目5个单项大奖也极有份量。

积极扩大消费。实现旅游总收入159亿元、增长12%；“冬交会”签约订单35.2亿元、增长33.8%；全年网签销售商品房383万平方米、增长29.8%，创历年最高。离岛免税销售增长36%。实施宽带网络提速降费，开展“互联网+”消费活动，电子商务交易额增长13%。万达、远大、吾悦等商业综合体进入投资高峰期，城市商圈进一步提升。

强化经济运行调节。建立经济运行月度分析制度，及时出台推进重点项目、加快产业发展、扶持中小企业等一系列措施，坚持每季有主题、每月有部署、每周有安排有督查有通报。抢抓政策机遇，打通安置房与商品房通道，实现投资和消费双拉动。争取中央、省级资金130多亿元，落实积极的财政政策。搭建政银企对接平台，推动金融机构与企业达成698亿元融资授信，帮助中小微企业解决担保贷款18亿元。推行“政保贷”、“助保贷”改革，累计注入政府风险补偿金2.4亿元、撬动社会资本25亿元。全市金融机构贷款余额比年初增长26%。

（二）全面开启“双创”模式

用“愚公移山”之志攻坚克难，举全市之力推进“双创”工作。坚持高位推进、高速运转，坚持问题导向、创建惠民，坚持营造氛围、突出内涵，坚持严管重罚、强势问责，坚持着眼长效、创新机制，坚持省市联动、全民参与，汇聚省直部门、中央驻琼机构、驻市军警、省属院校、社会团体、广大企业和市民等方方面面的力量，形成了全社会同心协力促“双创”的工作大格局，迅速实现了市容市貌、群众态度、干部作风“三大转变”。先后打响了小街小巷改造、交通秩序整治等九大战役，全市落实“门前三包”主体责任单位（业主）4.3万家，签约率100%；完成91条道路和12个小游园绿化；拆除占道疏导点115个，取缔马路市场66个；完成858条小街小巷改造、52条道路修复，启动46家农贸市场环境秩序整治和改造升级；集中整治停车场1079家，划设临时停车泊位3500个；查处交通违法39万件。实施“三园合一”，重点推进美兰机场互通、火山口大道等绿化景观提升工程，完成了滨海大道海岸线灾后修复等一批重点项目。大力培育和践行社会主义核心价值观，全民公益和志愿服务渐成常态，凡人善举层出不穷，崇德向上蔚然成风，全国道德模范提名奖9人，省道德模范29人，涌现出洪庆芝、蔡建家、黄良辉等一批先进典型。中央文明办调研组充分肯定了我市创建工作成果，《人民日报》、中央电视台、《海南日报》、海南广播电视总台等主流媒体均做了深度报道。

以“三严三实”为核心要求的“双创”模式，是我市历史上范围空前、力度空前的社会总动员，释放出磅礴正能量，不仅大大提升了城市管理治理水平，而且强力助推了各项事业的大发展。市民游客纷纷点赞，普遍认为海口形成了前所未有的人心思进、人心思干的良好氛围。

（三）加快产业转型升级

落实省委、省政府关于发展“十二大产业”、“六类园区”和“百个特色产业小镇”部署，出台互联网、金融、旅游、电商、房地产等产业引导政策，促进产业提质增效。

现代服务业快速发展。第三产业增加值增长9.7%，对经济增长贡献率达81%。旅游、会展、文体等产业进一步融合。冯小刚电影公社南洋街开街，中山路骑楼老街业态调整完毕，丽星邮轮以海口为母港开通航线。全市接待国内外过夜游客1220万人次、增长8%。全年举办规模会展活动223场，获评“中国会展名城”。金融业增加值增长30%，飞机保税融资租赁业务实现零的突破，海南银行落户海口，华夏银行海口分行开业，7家企业在“新三板”挂牌。新增注册互联网企业974家，60多家企业入驻复兴城互联网创新创业园，165家企业入驻江东电商产业园，阿里巴巴、乐视体育等落户海口，南海云及大数据服务中心运行，互联网产业聚集效应初显。林安智慧物流园开工建设，喜盈门综合物流商贸项目完工。

热带特色农业稳步发展。桂林洋国家热带农业公园开工建设，海口农副产品交易配送中心建成，罗牛山10万头现代化养猪基地完工，6家农业生产基地获评首批省级现代农业示范基地（园区）。改造标准化菜田1.5万亩，新增叶菜3000亩，全年蔬菜、冬季瓜菜、热带花卉种植面积分别为42.5万亩、20万亩和6.7万亩。新建畜禽标准化生态养殖小区21个，水产养殖产量增长14.3%。5个农产品获省名牌产品，7个农产品获省著名商标，新认证11个无公害农产品，云龙淮山、永兴荔枝获“国家地理标志产品”。

新兴工业进一步发展。康迪10万辆电动汽车、光启科学临近空间产业园等项目落地，先声药业、威特电气等项目开工建设。新增高新技术企业18家，总量占全省75%。与海马集团签约设立10亿元产业基金。落实工业企业扶持资金1.3亿元。美安科技新城基础设施累计完成投资近40亿元，“新药谷”加快建设，医药行业总产值增长21.5%、占全省97%。海口高新区工业总产值增长23%。

特色产业小镇建设积极推进。按照“一镇一品、各具特色”的原则，完成10个特色产业小镇发展规划，石山镇互联网农业小镇建设成效明显，演丰镇成为全省唯一的国家新型城镇化综合试点镇。2个村庄、18家“农家乐”分别被评为中国乡村旅游“示范村”和“金牌农家乐”。

（四）全力推进改革开放

快速推进三大重点改革。开启“多规合一”改革，完成《“多规合一”总体规划》、《“海澄文”一体化基础设施规划》编制，明确城市规模边界、生态保护红线、资源利用上限和环境质量底线，实现了目标、坐标、指标“一张蓝图”全方位统筹，推动解决了一批项目建设的历史遗留问题。深入推进城市管理综合执法改革，加大联合执法力度。深化行政审批制度改革，在上年审批时间压缩70%的基础上，再压缩审批时间19%、削减审批总环节17%。美安科技新城“极简审批”获得中央改革办和省委、省政府的充分肯定。市区镇（街）行政管理体制改革成效显著，坚持基层导向，着力推动人、财、物向基层倾斜，精简下放市级行政编制170名、事业编制765名，分别占编制总量的10%和11.6%，定额补助各区1亿元，解决了村（居）“两委”工作经费不足和基层干部待遇偏低问题，村（居）运转保障经费跃居全省前列。

统筹推进其他领域改革。投融资体制改革成果丰硕，成立海口投资管理有限公司，设立城乡发展股权投资基金、首期到位40亿元。PPP项目储备44个、投资额716亿元，入选全国第二批PPP示范项目数量全省第一，南渡江引水和琼山、龙华环卫一体化等PPP项目正式实施。深化商事制度改革，实施“三证合一、一照一码”，新增市场主体4.2万户、增长35.2%，注册资金增长21.4%。设立市公共资源交易中心。财政、不动产登记、国资国企、机关事业单位养老保险、储备粮管理等领域改革取得新进展。

加大对外开放力度。海陆空立体大通道基本形成，美兰机场旅客吞吐量突破1600万人次，港口过海旅客1330万人次、高铁接送旅客1150万人次，海陆空三大交通枢纽全年客流量超过4000万人次。与蒙古乌兰巴托、韩国釜山海云台结为友城，与马来西亚巴生港缔结友好港。海口港被列为国家“一带一路”15个沿海港口之一，“海口—厦门”等集装箱航线开通。汽车整车进口口岸投入运营。海航集团进入世界500强，海马汽车、立昇净水布局海外。成功举办“中国海南21世纪海上丝绸之路”电影节、世界自然保护联盟第87届全球理事会、中国国际商标品牌节等一批重要国际国内活动。先后组织参加央企战略合作座谈会、“厦洽会”等经贸招商活动，签约金额820亿元。

（五）生态环境持续改善

坚持生态立市，坚持以生态领规划，坚持城市开发边界避让生态红线。加强大气环境治理，责令83家违法排放企业停产，淘汰黄标车1.7万辆，新投放环保公交车217辆，清洁能源公交车比例提高至93%，空气质量在全国74个重点城市中稳居第一。开展全市18个水体治理，完成白沙门污水处理厂（一期）升级改造，南渡江龙塘段、永庄水库等饮用水源地水质达标率100%。全面开展海岸带保护与开发专项检查，组织拆除违章建筑20宗。深入实施“绿化宝岛大行动”，植树造林3.6万亩。加强农业面源污染、违规采砂等专项整治，大力开展东寨港红树林保护区周边环境治理。保持“打违”高压态势，集中清理领导干部和公职人员及其直系亲属参与违法建筑的行为，向社会公布受理短信和微信举报号码，全市拆除违建366.4万平方米、防违控违67.3万平方米。

（六）全力保障改善民生

全年民生支出128.4亿元、增长25.2%，占地方公共财政支出的75.1%、同比提高5.1个百分点。

全面完成民生实事。新增中小学学位5050个。新开通和调整优化公交线路12条，海甸岛环岛路贯通，海甸五西路、海彤路改造完工。完成道路积水点改造8处。建成街心小游园8个。出台城市水体保护蓝线规划。确认及办理困难群众户口登记2000人。解决房地产办证历史遗留问题1203户、20.7万平方米。改造农村危房4300户、新建防洪楼11栋、建成7个镇农村生活垃圾收运体系，新增农村有线电视用户6512户、农村广播站300个。27个村（居）社区活动中心、11间农村老年人日间照料中心投入使用。完成菜篮子公司筹建，持续扩大叶菜种植，优化流通体系，设置17个连锁超市销售专区、25个社区销售点和50家农副产品平价商店（专区），延长平价菜供应3个半月，多措并举保供稳价。涉及海口的省政府为民办实事事项全部完成。

加快发展社会事业。新增城镇就业5.7万人。城乡居民养老保险参保31.2万人，参保率97.6%。城乡居民养老保险基础养老金标准提高到145元，城市低保、农村低保标准分别提高到520元和460元。城镇居民医保参保56.5万人，完成年度任务的111%。145个贫困村全部脱掉“贫困帽”。完善农村安全饮水工程99处，受益人口11.4万人。配售配租保障性住房2370套。省肿瘤医院、海南新现代妇女儿童医院投入运营。所有公立医疗机构实施“先看病、后付费”，镇级医院全面推行“限费医疗”。实施单独两孩政策，出生人口性别比控制在目标范围内。为6447对怀孕夫妇开展国家免费孕前优生健康检查和地中海贫血筛查诊断。为困难群众和农民工提供法律援助9024件，挽回经济利益1.8亿元。新建35个行政村文化活动室。《海口晚报》更名《海口日报》暨“海口发布”正式上线。海口代表团在第一届全国青运会夺得1金3银，并获体育道德风尚奖。

狠抓社会综合治理。积极构建立体化治安防控体系，深入开展“雷鸣行动”、“飓风禁毒”等专项行动，严厉打击各类违法犯罪活动。调处各类矛盾纠纷8100多宗，成功率97.5%。强化食品药品监管和质量技术监督，安全生产形势保持总体稳定。防灾减灾体系不断完善，连续三年被评为全国地级市防震减灾工作综合考核先进单位。军民融合发展，国防动员、人民防空、优抚安置扎实推进，连续第八次获得“全国双拥模范城”荣誉称号。统计、气象、保密、档案、史志、民族宗教、外事侨务、对台事务等工作取得新成绩，工会、青少年、妇女儿童、老龄、残疾人、慈善等事业取得新进步。

（七）政府效能明显提升

认真贯彻中央、省委省政府要求和市委部署，切实加强政府自身建设。深入开展“三严三实”专题教育，严格执行中央八项规定、国务院“约法三章”、省委省政府二十条规定和市委二十一条规定。全年处理“庸懒散奢贪”、“四风”问题117起468人，“三公”经费下降17%。及时向人大和政协报告工作、通报情况，广泛听取各民主党派、工商联、无党派人士的意见建议。提请市人大常委会审议法规议案3件，制定政府规章4件、废止1件，获省依法行政考核“七连冠”。办理人大代表建议288件、政协委员提案368件，办结率均为100%。积极推进法治政府建设，加强审计监督，规范完善重大决策、重大项目安排、大额度资金使用决策议事规则，8家单位开展权力清单和责任清单试点。深入开展“学乐东，见行动”活动，发扬“四种精神”，着力抓好“天大的小事”，打造新型问政平台《亮见》，进一步推动作风转变，行政效能不断提升。

各位代表！2015年各项目标任务的胜利完成，确保了“十二五”圆满收官。五年来，我们坚持在省委、省政府和市委的坚强领导下，在市人大、市政协的监督支持下，成功应对经济下行压力，成功战胜“威马逊”、“海鸥”等自然灾害，成功克服电力短缺、持续高温等不利影响，爬坡过坎，砥砺奋进，经济社会发展取得了重大成就。

过去的五年，经济发展迈上新台阶，省会城市首位度进一步提升。地区生产总值、全社会固定资产投资双双突破千亿大关，地方一般公共预算收入跨上百亿台阶，农村常住居民人均可支配收入过万元，人均地区生产总值、城镇和农村常住居民人均可支配收入高于全国平均水平。与“十一五”末相比，地区生产总值增长59.7%、地方一般公共预算收入增长121%、全社会固定资产投资总额、社会消费品零售总额分别增长189%、82%，城镇和农村常住居民人均可支配收入分别增长72%、83%。2015年地区生产总值、全口径一般公共财政收入、全社会固定资产投资分别占全省比重31.5%、29.3%和30.4%，比“十一五”末分别提升1.8个、4.9个和4.1个百分点。

过去的五年，基础设施投入持续加大，城乡面貌明显改善。完成基础设施投资985.4亿元，是“十一五”的1.9倍。城市建成区扩大到151.6平方公里，累计完成棚户区改造2.5万户、惠及8.7万人。秀英港区搬迁加快，环岛高铁在海口闭环，美兰机场一期扩建完成，定海大桥、新东大桥完工，铺前大桥开工建设，12米以上市政道路达796公里，初步搭建了海陆空互联互通的总枢纽交通体系。累计建成农村公路4200公里，农村生产生活条件逐步改善。

过去的五年，重点领域改革深入推进，对外开放程度不断提高。纵深推进强区扩权，开展计划单列镇试点，深化新一轮市区财政体制调整。多轮清理市级行政审批事项，精简事项713项、降幅在70%以上。完成社区网格化管理体制改革。国资国企、教育文化、事业单位等重点领域改革都取得了新进展。国际旅游岛优惠政策进一步落地，保税贸易快速发展。内外贸海运航线30条，国内外航空航线200条、通达102个城市。主动融入“一带一路”、泛珠区域和北部湾合作，成功举办一批国际性会议和赛事活动，国际友城达30个，海口知名度和影响力不断提升。

过去的五年，生态文明建设富有成效，生态环境质量保持优良。强化生态立法保护，东寨港红树林湿地保护范围从5万亩扩大到12万亩。绿化覆盖率、森林覆盖率分别达43%和38%。实施水环境治理、大气治理、农村生活垃圾无害化处理等重大环保工程，城市污水集中处理率达92%，全市集中式饮用水源地达标率100%，城市生活垃圾无害化处理率100%，空气质量优良率保持在98%以上。坚决杜绝低水平、消耗资源、重复建设的项目进入，完成“十二五”节能减排目标。

过去的五年，社会事业快速发展，人民生活水平稳步提高。坚持兜底线、补短板，民生支出五年累计475亿元，是“十一五”的2.8倍。农村贫困人口减少1.4万人。累计新增就业25.9万人，农村富余劳动力转移就业5.9万人。城镇保障性住房覆盖面达到38%。城乡社保制度全覆盖，企业退休人员养老金和城乡居民养老保险基础养老金标准不断提高。实施医药卫生体制改革，药品零差率销售、病种限价等工作深入推进。坚持教育优先发展，实施第一轮学前教育三年行动计划，大力整合城区麻雀学校，完成“校安工程”301个，累计增加学位2.8万个，龙华区、美兰区、琼山区通过义务教育发展基本均衡县（市、区）国家验收。

各位代表！回顾“十二五”的历程，我们深切地感受到，这五年，全市经济社会发展跃上新台阶，城乡面貌发生显著变化，人民群众获得感明显增强。这些成绩的取得，得益于省委、省政府和市委的坚强领导，得益于市人大、市政协及各位代表、各位委员的监督支持，得益于历届“四套班子”打下的坚实基础，也是全市上下团结一心、拼搏实干的结果。五年来，全市广大干部以发展大局为重，以人民福祉为重，敢于担当，尽心尽力，用自己的辛苦指数换来了群众的幸福指数、企业的满意指数和海口的发展指数！在此，我谨代表市人民政府，向全市人民，向人大代表、政协委员、各民主党派、工商联和社会各界人士，向驻市部队、武警官兵和公安司法干警，表示崇高的敬意和衷心的感谢！

“十二五”发展成就令人振奋。同时，也要清醒地看到海口经济社会发展还存在不少问题：实体经济不够发达，产业科技含量不高，创新驱动能力不强；投资结构不够合理，生产性项目比重不高；基础设施和公共服务历史欠账较多，民生事业还有不少短板；城市治理管理有待进一步加强，城乡发展不够平衡；人才资源比较匮乏，创新创业氛围不够浓厚；对外开放水平不高，体制机制活力有待进一步释放；一些部门忙闲不均，个别干部对群众态度冷漠，不作为、乱作为、不善为和“庸懒散奢贪”等现象仍不同程度存在。对这些问题，我们将高度重视，认真解决。

二、“十三五”发展总体思路和目标任务

“十三五”时期，是海口全面建成小康社会的决胜阶段。从全国看，我国经济韧性好、潜力足、回旋空间大、经济长期向好的基本面没有改变。从全省看，面临国家“一带一路”、“海洋强国”、“创新驱动”、“互联网+”等发展战略机遇，海南国际旅游岛建设发展前景广阔。从自身看，“多规合一”试点全面实施，“海澄文”一体化综合经济圈启动建设，经济综合实力和抗风险能力不断提高，城市基础设施日臻完善，区位、政策、生态等优势进一步彰显。特别是全面开启“双创”模式后，全市上下政通人和、务实担当，广大干部精神焕发、劲头十足。总的来看，未来五年，海口处于发展的重要机遇期、上升期，完全有条件、有能力实现更好、更快的发展！这既是我们肩负的历史使命，又是全市人民的共同期盼！

“十三五”发展的指导思想是：高举中国特色社会主义伟大旗帜，全面贯彻党的十八大、十八届三中、四中、五中全会精神，以马克思列宁主义、毛泽东思想、邓小平理论、“三个代表”重要思想、科学发展观为指导，深入贯彻习近平总书记系列重要讲话精神，紧紧围绕“四个全面”战略布局，牢牢把握发展第一要务，全面贯彻“创新、协调、绿色、开放、共享”的发展理念，抢抓“一带一路”倡议重大机遇，加快形成引领经济新常态的体制机制和发展方式，充分发挥生态环境、经济特区、国际旅游岛优势，以生态领规划、以项目保增长、以棚改促建设、以“双创”抓管理、以真心惠民生、以铁腕转作风，统筹推进经济建设、政治建设、文化建设、社会建设、生态文明建设和党的建设，努力打造“21世纪海上丝绸之路”战略支点城市、大南海开发区域中心城市、全国生态文明示范城市和海南“首善之城”，率先在全省全面建成小康社会，当好海南争创中国特色社会主义实践范例、谱写美丽中国海南篇章的排头兵。

今后五年经济社会发展主要目标是：经济保持中高速增长。地区生产总值年均增长8%，到2020年人均地区生产总值进入中高收入阶段；地方一般公共预算收入年均增长8%；全社会固定资产投资至少翻一番，生产性项目投资占比50%以上。户籍人口城镇化率达到51%。现代服务业支撑作用进一步增强，高新技术产业和低碳制造业比重进一步提高，农业现代化取得突出进展，形成具有地方特色、具有较强竞争力的现代产业体系。人民生活水平和质量普遍提高。城乡居民收入增长与经济发展同步，力争达到全国中上水平。物价水平保持在合理区间，基本公共服务均等化水平走在全省前列，率先实现城乡公共服务一体化。城市文明程度和市民素质显著提高。社会主义核心价值观深入人心，建成全国文明城市和国家卫生城市，力争成为全国首批健康城市。生态环境质量始终保持一流水平。生态文明制度更加健全，资源环境容量与综合承载能力明显增强，生态环境质量始终走在全国前列。改革开放和依法治市取得新进展。若干领域改革取得重要突破，开放型经济加快发展。政府职能进一步转变，社会治理体系和治理能力法治化、现代化水平明显提高。

各位代表！今后五年，我们坚持稳中求进的总基调，重点把握好以下五个方面：

（一）坚持创新发展，提升发展质量和效益，着力构建创新引领的经济体系和发展模式。实施“人才强市”战略，推进大众创业、万众创新。加快推进“海澄文”一体化综合经济圈建设，积极申报海口国家级新区，力争到2020年三市县地区生产总值占全省50%以上、旅游收入占全省25%以上。推动供给侧改革，紧紧围绕全省十二大重点产业，加快产业转型升级，加快发展旅游、金融保险、文化体育、医疗健康、互联网、商贸物流等现代服务业，促进热带特色现代农业规模化、标准化、绿色化、品牌化，推动高新技术产业和低碳制造业发展。着重建设“六类园区”，发展“飞地经济”。

（二）坚持协调发展，强化统筹兼顾，注重发展的整体性和均衡性，在加强薄弱领域中增强发展后劲。坚持经济社会发展与城市建设管理并重，统筹推进“五网”基础设施建设，同步推进旧城改造与新区建设，实现城市风貌、城市管理大变样。坚持城乡一体化发展，深化户籍制度改革，加快构建“主城区—特色产业小镇—建制镇—美丽乡村”四级城镇空间结构。坚持陆海统筹发展，提升海洋经济发展水平，积极发展海洋交通运输业、海洋旅游业、海洋新兴产业，着力打造南海资源开发和服务保障基地。坚持经济建设和国防建设融合发展，深入开展“双拥共建”活动，加强应急专业和保障力量建设，增强军地联合应对处置各类突发事件的能力。

（三）坚持绿色发展，精心呵护得天独厚的生态环境，形成人与自然和谐发展现代化建设新格局。落实生态空间布局，设定并严守资源消耗上限、环境质量底线、生态保护红线，对重大生态责任事故“一票否决”。实施治污工程，开展排水管网雨污分流改造，加强大气、水、土壤及固体废弃物污染防治和农业面源污染治理。建设热带滨海花园城市，做好顶层设计，抓好城乡造林绿化，深入实施“300米见绿、500米见园”工程、“增荫添彩”工程、园林精品工程和立体绿化工程，提高城市“绿视率”、“花视率”。建立绿色低碳循环发展产业体系，实施能耗强度和总量双控，大力推广清洁能源和新能源应用，倡导绿色出行，大力发展城市公共交通。集约节约利用资源，实行最严格的水资源管理和节约用地制度，提高重点开发区域单位土地投资强度和产出效率。

（四）坚持开放发展，全力提升对外开放水平，建设“21世纪海上丝绸之路”战略支点城市。拓宽开放视野，积极融入“中国—中南半岛经济走廊”建设，主动参与环南海经济合作圈建设。全面提升交通内外互联互通水平，强化区域交通枢纽地位，积极推进海口—湛江的高速铁路融入国家高铁网，努力将海口港打造成区域性综合枢纽，将美兰机场打造成连接内陆与东南亚及洲际的国际航空转运枢纽。规划建设大容量快速公交和城市轨道交通系统，实现港口、高速公路、铁路、机场、城市公交的无缝对接。在产业园区复制和推广自由贸易试验区政策，加快开放型经济发展。扩大经贸文化交流与合作，密切与东盟各国在港口物流、产业合作等领域的往来，打造面向东盟国家的区域性总部基地。搭建对外经贸文化交流平台，探索与海上丝绸之路沿线国家互办文化年、旅游年。

（五）坚持共享发展，把发展成果更好体现在增进民生福祉上，着力打造全市人民的幸福家园。提升基本公共服务均等化水平，增加公共产品和公共服务供给。实施脱贫攻坚，力争2017年底现行标准下贫困人口全部脱贫。大力发展教育事业，全面推进各级各类教育协调发展，提高教育质量。鼓励支持社会资本兴办教育，加快桂林洋教育产业园建设。加强公共卫生和人口计生工作，推进“健康海口”建设，深化医药卫生体制改革，完善基层医疗服务模式，坚持计划生育基本国策。实施积极就业政策，构建和谐劳动关系，合理调整收入分配。实施全民参保计划，积极落实基本养老、基本医疗保险、失业保险、生育保险、工伤保险省级统筹。完善以政府为主提供基本住房保障、以市场为主满足多层次需求的住房供应体系。落实征地留用地政策。完善社会救助体系，健全城乡居民最低生活保障制度。大力实施“菜篮子”工程，让人民群众吃上“便宜菜”、“放心粮”。

三、2016年工作安排

2016年是“十三五”和全面建成小康社会决胜阶段的开局之年，是全面深化改革的攻坚之年，是“双创”工作的决战之年，做好今年工作至关重要。

今年主要预期目标是：地区生产总值增长8%—8.5%；地方一般公共预算收入增长9%；全社会固定资产投资增长30%左右；社会消费品零售总额增长8%；城镇和农村常住居民人均可支配收入分别增长8%、9%；居民消费价格指数涨幅控制在3.5%以内；城镇登记失业率控制在3%以内；完成省下达的年度节能减排任务。

各位代表，今年的目标是积极向上、自我加压、难度很大的目标。需要大力弘扬“四种精神”，需要坚持“双创”模式和动力，需要继续落实“百日大会战”的联动和督办机制。抓好今年的工作，要突出阶段主题，明确每月重点，建立长效机制，着重做好以下九个方面工作：

（一）加大有效投资力度，确保经济健康快速增长

全面实施“三个千亿”计划。坚定不移优化投资结构、扩大投资规模，确保全市83个省重点项目完成年度投资450亿元。一是突出抓好生产性项目。着力引进一批投资额在5亿元、10亿元、20亿、数十亿元以上的生产性项目落地，力争生产性项目投资占重点项目比重50%以上。二是加快“五网”建设。推进新海港区二期、马村港区三期、江东大道二期、定海大桥海口段连接线等项目建设。实现全市光纤宽带用户占比达到65%，90%的重点公共场所WiFi覆盖，建成区4G信号全覆盖。完成龙泉、江东等变电站建设，将燃气管道向镇墟延伸。建成地下综合管廊10公里、新建15.5公里，加快推进南渡江左岸片区农田排涝等工程建设。海秀快速路7月30日主桥通车、9月30日全面竣工。三是全力推进棚改工作。落实“棚户区改造三年滚动”计划，坚持棚改与改善群众生活、调整产业结构、加快城市更新、消化房产存量相结合，引进社会资本参与棚改，继续推行货币化安置，加快14个在建项目、新启动14个项目，不折不扣完成省政府下达的任务。

努力扩大消费需求。继续开展旅游、房地产、热带特色农产品“三大促销”，力争旅游总收入增长12%以上、网签销售商品房380万平方米以上。抢抓离岛免税政策放宽机遇，确保离岛免税消费高速增长。进一步放大整车进口口岸政策效应，推进进口商品直销中心和跨境电商平台建设，做大线上销售业务。顺应个性化、多样化、体验式消费需求，扩大文体娱乐、养生健康、信息等各类新兴消费。培育消费热点，改善消费环境，加快王府井奥特莱斯等大型商贸项目建设，深入推进电子商务发展。

（二）开展“双创”决战年活动，全面加强城市工作

全市上下必须凝心聚力，必须精神不变、意志不退、力度不减，坚决打赢“双创”攻坚战，决战2016、决胜2017。

强化整治巩固成果。城市管理与建设同等重要，建设要在管理中实施，管理要在建设中推进。依法持续开展城乡环境、道路交通、市容市貌、环境卫生、安全秩序、公共卫生等“六大治理”，巩固和深化“门前三包”责任制。健全覆盖城乡的网格化管理体系，加强“数字城管”建设。强化“三无”小区管理，推动各类住宅小区成立业主委员会。推进城市综合执法改革，打破部门行政执法职能分割，下沉执法资源和权限，实施以区、镇（街）为主的多部门综合执法。

深化创建提升素质。继续加强社会主义核心价值观宣传教育，深化文明村镇、文明家庭、文明单位、文明校园等创建工作，带动社会文明程度整体提升，引导群众从“要我创建”真正转变为“我要创建”。加快“诚信海口”、“公益海口”建设，建立健全社会诚信体系，推进公益工作和志愿服务常态化、制度化。坚持弘扬传统美德，彰扬善行义举，在全社会唱响主旋律、弘扬正能量。

提升城市景观风貌。加快城市主要门户和进出通道、重要公共开放空间等主要节点的绿化、亮化、美化、彩化。全面开展高铁海口段和绕城高速沿线环境整治。探索推行园林建养PPP模式，加快推进“三园合一”和主要干道景观提升工程，推进海绵城市建设。完成美舍河带状公园、15座人行天桥立体绿化工程，举办首届海南国际旅游岛三角梅花展，推进现代化热带滨海花园城市建设。

完善提升城市功能。进一步完善城区路网，继续打通断头路，完成改造主次干道路61条、小街小巷1576条。加快公交行业改革，推进停车场、公交场站建设，抓紧大容量公交系统项目前期工作。新建5家农贸市场。启动垃圾分类，新建８座小型生活垃圾收集站，完成餐厨垃圾处置和建筑垃圾再利用项目建设，确保垃圾综合处理二期投产运行。推进美兰、秀英环卫一体化PPP项目。继续加大道路积水点改造，健全城市救灾减灾体系。加快省儿童医院等民生项目建设，启动市级图书馆、美术馆“两馆”建设，抓紧区级文化馆、村级文化室建设，完善一批居民小区文体设施。

海口是海南唯一的大城市。只要坚决贯彻中央城市工作会议“五个统筹”精神，以“双创”为动力，举轻若重，落细落小，久久为功，建设幸福美丽、和谐宜居海口的目标就一定能实现。

（三）大力发展实体经济，努力提高有效供给

实施精准产业政策。针对旅游文体、金融会展、互联网、交通物流、医疗健康、特色农业、低碳制造等重点产业，研究制定更积极、更精准的政策。鼓励大企业在海口注册公司或设立总部机构，积极培育发展总部经济、楼宇经济。对符合产业导向的重点项目加强资源倾斜，实施“一企一策”。清理整合原有产业引导资金，优化使用方向，加强投入绩效评估。引进社会资本设立产业投资基金，支持符合政策导向的企业申请国家和省里的资金。落实降低实体经济企业成本行动，清理和规范涉企收费，切实减轻企业综合性成本负担。

加快发展现代服务业。落实“国际旅游岛+”部署，培育壮大邮轮游艇、特色街区、低空飞行等旅游新业态，启动5A景区创建，加快冯小刚电影公社老北京街、长影环球100、热带野生动植物园等项目建设，确保观澜湖水世界投入运营，推动“旅游城市”向“城市旅游”转变。支持金融业务创新，大力发展普惠金融，努力打造金融机构集聚区。加快文体产业发展，促进国家足球训练基地落地，推进五源河文体中心、永兴体育小镇等项目建设，培育发展帆板帆船、马拉松等品牌体育赛事，加快骑楼、琼山府城历史街区和海瑞墓、五公祠等文化古迹保护修缮。打造一批知名会展品牌，力争全年会展综合收入增长10%。落实“互联网+”行动，继续引进一批互联网龙头企业，支持本土互联网企业做大，积极培育软件、游戏动漫、服务外包等产业，充分发挥南海云及大数据服务中心平台功能，做大互联网产业规模。加快现代物流业发展，启动综合服务、生产服务、商贸服务等物流园区规划布局，依托椰海大道打造商业物流走廊，推进美安物流园等项目建设。加大房地产结构调整力度，优化空间布局，努力扩大有效需求。

大力发展医疗健康产业。充分利用高端医疗资源集聚和城市配套齐全等综合优势，全力打响海口医疗健康品牌。支持社会资本与国内外知名医疗机构创办综合性医院，年内力争1—2家高端医疗机构入驻。推广“医养”结合模式，鼓励医疗与养老机构建立业务合作机制。积极发展健康检测、调理康复等健康管理服务产业。支持发展商业健康保险，落实买商业健康保险抵个人所得税试点政策。推进房地产、旅游等产业与医疗健康产业互动发展。支持引进一批高端医疗人才和学科带头人，支持高校和职业学校开设健康服务专业，加快培养医护、康复等专业人才。研究推进国际健康生态城项目建设。

做实做优低碳制造业。力争康迪10万辆电动汽车2月份开工，光启科学临近空间产业园年底完成基建工程，先声药业等项目竣工投产，确保“新药谷”医药健康产业园取得实质性突破。推进中心城区工业企业向园区聚集，进一步放大产业聚集效应。实施创新驱动战略，加快以企业为主体的科技创新体系建设，开展产学研合作，建立技术研发中心。力争新增高新技术企业6家以上，全年规模以上工业增加值增长7%。

加快热带特色农业转型升级。按照“三区一中心”定位加快桂林洋国家热带农业公园建设，确保一期年底开园。依托罗牛山农产品加工园发展精深加工，提升农产品附加值。创建一批省级现代农业示范基地，推进羊山石斛、文山沉香、大力洋果蔬、三江莲雾等产业园建设。继续抓好冬季瓜菜、常年蔬菜基地建设，新认定无公害农产品基地3000亩，打造10个省级知名品牌。新建一批标准化畜禽养殖小区，大力发展循环农业。进一步优化种养结构，积极培育新型经营主体，大力发展涉农电商，支持开展“科技下乡”。

大力发展海洋经济。坚持陆海统筹，加快南海资源开发和服务保障基地建设，力争在海洋港口运输、海洋生物制药、海洋服务业等方面引进一批重大项目。支持丽星邮轮以海口为母港开辟新航线，加快南海明珠国际邮轮中心建设，推进如意岛等生态岛屿建设。积极引进海洋研究机构、重点实验室落户。

（四）打响脱贫攻坚战，统筹城乡建设

精准脱贫攻坚是重大政治任务，是重大民生工程、德政工程。决不能让一名贫困群众掉队，全面小康属于所有海口人民。

实施精准脱贫攻坚。实施“两年脱贫、三年巩固提高”计划，以“三严三实”作风为保障，提前实现贫困人口全部脱贫。一是精准确定对象。准确掌握贫困户的基本情况、动态信息，建档立卡。注重引导贫困群众转变观念，充分调动扶贫对象的积极性、主动性，激发内生动力。二是精准滴灌施策。综合采取产业发展、乡村旅游、电子商务、劳务输出、教育文化、卫生健康、科技人才、基础设施建设、生态补偿、社保政策兜底等“十大措施”，因人因户制宜，定向施策脱贫。三是精准制定政策。用好用足中央、省级政策，结合市情完善扶贫政策。加大扶贫投入，捆绑使用涉农资金、扶贫资金，免费开展创业和实用技能培训，给予生产贷款贴息，着重改善生产生活条件，增强自我“造血”功能。四是精准考核验收。出台扶贫开发工作考核办法，逐户定时间、定任务、定措施、定人员、定成果，严格评估验收，严格脱贫销号退出，切实做好脱贫巩固工作。五是精准落实责任。严格落实一把手责任制，市、区、镇、村四级主要领导一起抓，脱贫责任落实到具体人，一对一挂钩结对扶贫，层层压紧压实责任。广泛动员社会力量参与，构建政府、市场、社会协同推进的大扶贫格局。

加快新型城镇化。推进石山、新坡、云龙、大致坡等10个特色产业小镇建设，强化镇域产业支撑。启动实施镇墟基础设施建设三年行动计划，完善环卫、排污和公共服务等配套建设，建设宜居小镇。结合全省“千个美丽村庄”建设，整合资源资金，集中建设一批生产发展、村容整洁、设施齐全、乡风文明的农村新社区。加快建设小微水利、农村公路、电力通讯等基础设施，实施自然村“村村通”工程，新建364座分散式和10座集中式供水工程，加快南渡江引水、桂林洋防潮堤加固等工程建设。积极推行农村“一池三改”。依法依规推进农村土地有序流转，解决部分农村土地撂荒问题，发展壮大村集体经济。继续推进文明生态村建设。

（五）进一步深化改革，激发内生动力活力

全面实施“多规合一”改革。坚持“一张蓝图干到底”，建立“多规合一”编、审、管、控体制机制，探索推进“多规合一”综合执法改革和城市管理综合执法改革“合二为一”。完成数据库和应用平台搭建，促进行政审批制度改革。根据《“多规合一”总体规划》，强化城市设计，全面启动主城区控制性详规、镇域控规和村庄规划修编。

纵深推进行政管理体制改革。理顺市、区两级政府之间的权责划分，优化部门内设机构设置及业务流程，加快推进事业单位分类改革。推广“极简审批”试点经验，将权力清单和责任清单编制扩展到所有行政部门。推进新版行政审批系统上线，力争“一张审批网”市、区、镇和村四级覆盖率60%以上、网上审批比例80%以上。坚持固本强基，坚决推进资金下沉、权项下放、人员下派，实施市级部门瘦身、减编、下沉，做实做强区级和镇（街）级，解决全市230个村（居）办公活动场所问题。

统筹推进财政、投融资等领域改革。合理划分市、区事权和支出责任，推进专项资金整合统筹，抓好支出绩效考核，强化政府债务管理。落实国税地税征管体制改革。深化投融资改革，积极推广政府和社会资本合作模式。完善国有资产管理体制，深入推进国企改革试点。积极配合全省农垦管理体制改革，落实农场属地化管理。实行居住证制度，稳步实施户籍制度改革。推进农业农村、不动产登记等领域改革。

（六）落实“一带一路”倡议，扩大对外开放

充分发挥中心城市辐射带动作用。按照“大发展、大开放、大合作、大生态”理念，加快“海澄文”一体化综合经济圈建设，务实推进资源利用、产业发展、基础设施、生态环保、公共服务、配套政策“六个一体化”。推进绕城高速二期、海文高速与绕城高速连接线建设，提前谋划城际轨道交通、琼北滨海旅游公路等项目。积极推动“海澄文”基础设施一体化投融资平台建设，探索三地互建“飞地园区”。推进美安科技新城与老城经济开发区的基础设施互联、产业发展互补。启动美兰临空产业园、马村临港产业园建设。

加快构建海陆空立体大通道。继续深化“三地七港”合作，争取省里支持海口港整合全省“四方五港”资源。培育和加密沿海港口及东南亚航线，增强海口港国际中转服务功能。提高美兰机场航空运输中转能力，打造连接内陆与东南亚及洲际的国际航空转运枢纽。做好新海港区、美兰机场、高铁海口东站三大“零换乘”枢纽前期工作。

积极扩大对外合作交流。加大与“21世纪海上丝绸之路”沿线国家的经贸、文化和人文交流，加强友城合作。服务保障和利用好博鳌亚洲论坛年会、全国旅游工作会议，继续举办“海口—东盟国家驻广州总领馆对话会”。推进与泛珠三角、北部湾等区域合作。加快发展保税贸易、跨境电子商务，设立国际大宗商品交易展示平台。支持企业实施“走出去”战略。办好中国（海南）海上丝绸之路文化产业博览会等知名会展。切实加大招商引资力度，建立招商常态机制，紧扣高端制造、科技研发等生产性项目开展定向招商，真正引进一批投资强度大、税收贡献高、就业容量大的项目。全面落实与省属国有企业战略合作协议，继续深化与本土企业合作。

（七）开展专项整治行动，巩固提升生态优势

大力整治违法建筑。保持“零容忍”高压态势，进一步强化区、镇、村三级打违主体责任，坚决杜绝出现新的违建，完成存量违建处置。加强源头防范，全面开展城乡居民宅基地确权颁证，实施农村建房报建管理。

深入开展水体、大气、土壤等专项整治。继续整治市区内河（湖）水污染，开展河、湖、沟清淤疏浚，重点治理18个水体。完成永庄水库饮用水源地一级保护区征地和隔离防护工程。新建扩建桂林洋、江东等污水处理厂。全面实施大气污染治理，确保空气质量在全国74个重点城市中继续保持第一。重拳打击违法排污等行为，加强耕地修复与治理。深入开展东寨港红树林退塘还林，启动和加快三江、玉龙泉、沙坡—白水塘等湿地公园建设。

建立健全生态保护体制机制。探索区域环保治理联防联控，积极推行环境污染第三方治理，建立多元化资金投入机制。争取将东寨港红树林保护纳入国家生态补偿转移支付范围。加强生态环保宣传教育，推进生态环境监测网络建设。严格落实节能减排，大力发展循环经济，推行清洁生产，实现各类资源节约高效利用。全面完成省下达的年度节能考核目标。

（八）保障改善民生，加快社会事业建设

坚持“可持续、保基本”原则，增投入、补短板，兜底线、织密网，压缩一般性开支，确保民生投入。

办好十件民生实事。精准减贫4532人，巩固减贫4485人；建设农村公路200公里；增加中小学学位6000个以上；建设行政村文化室40个；改造升级农贸市场46家；新开通和优化公交线路12条以上，建设智能公交示范工程（一期）；建设10个小游园和街边绿地；改造公厕36座；实施市政道路照明拓展工程；改建残疾人就业服务中心。

完善社会保障体系。积极开发公益性岗位，确保城镇“零就业”家庭和失地农户至少有一名适龄劳动力实现就业。加强就业技能培训，年内培训农村劳动力3000人，实现新增城镇就业4.5万人。开展全面参保登记，推进社保征缴扩面，全面推行社会保障卡应用。整合城乡居民基本医疗保险制度，完善居民大病保险政策。巩固城乡居民基本养老保险制度整合成果，推进社会化养老服务试点。推动公共租赁住房、廉租住房和“双困房”并轨运行。充分发挥菜篮子集团作用，新建1-2家大型农产品批发市场，畅通流通体系，努力保供稳价。落实国家储备粮增储计划。完善社会救助和保障标准与物价上涨挂钩的联动机制。

发展社会事业。继续抓好公办幼儿园、北师大合作办学、“10+2”工程和教育信息化等项目建设，确保五源河学校建成招生。加快发展职业教育，支持和规范民办教育发展。秀英区通过义务教育发展基本均衡县（市、区）国家验收。深化医药卫生体制改革，强化镇村卫生一体化管理，探索推进分级诊疗制度，提高城乡医疗资源利用率。实施一对夫妇可生育两个孩子政策。继续实施文化惠民工程，开展群众性文艺活动，打造一批文艺精品。保护和传承琼剧等非物质文化遗产。

强化社会治理。推进“平安海口”建设，完善立体化社会治安防控体系。完善重大决策社会稳定风险评估机制和社会预警体系，提高应急管理水平。强化产品质量和食品药品安全监管，落实安全生产责任，预防和遏制重特大安全事故。巩固和创新“法律六进”活动，扎实开展宪法和法律宣传教育，加强社区矫正工作。深化信访工作改革，推进法治、阳光、责任信访。坚持军民融合发展，支持军队和国防建设。继续做好统计、气象、档案、史志、保密、外事侨务、对台、民族宗教等各项工作，充分发挥工会、共青团、妇联、残联等人民团体桥梁纽带作用。

（九）加强政府自身建设，优化发展环境

坚守政治纪律，严守法纪规矩，努力建设廉洁高效法治诚信政府，营造风清气正的经济社会发展环境。

打造法治廉洁政务环境。自觉接受人大及其常委会工作监督、法律监督和政协民主监督，广泛听取民主党派、人民团体和各界人士的意见建议，主动接受司法监督和社会公众监督。严格落实党风廉政建设制，坚决履行和压实主体责任，坚持“一岗双责”，持续加强和改进作风。严格按照法定权限和程序履行政府职能，严格落实行政执法责任制和执法过错责任追究制。严控“三公”经费，完成公车制度改革。强化对行政审批、招投标、土地出让、政府采购等重点领域和关键环节的监督。坚决整肃庸政懒政怠政，营造敢于担当、主动作为的干事创业氛围。

打造高效便捷服务环境。开展“行政效能提升年”活动，建立健全政策落实的责任机制、奖勤罚懒的激励机制和常态化的督查问责机制，从严查处各类“中梗阻”行为。推广“双随机”等监管方式，严格窗口单位管理，强化网上政务服务中心和实体服务大厅建设，为项目建设和群众办事创业提供便捷高效服务。继续开展重点项目“服务月”、“督查月”等系列活动，实行重点项目跟踪联系协调制度，解决项目建设困难，完善项目落地配套条件。

打造吸引人才社会环境。落实“人才强市”战略，研究出台鼓励人才引进、培养、使用的政策措施，努力造就结构好、能力强、素质高的人才队伍。着力解决人才后顾之忧，在住房保障、户籍迁移、家属就业、子女入学等方面提供全方位服务。推进创业孵化基地、青年创业服务中心等平台建设，鼓励风投、创投等基金入驻，支持创新创业。

各位代表！海口正站在一个新的历史起点上，任务艰巨，使命光荣。让我们更加紧密地团结在以习近平同志为核心的党中央周围，在省委、省政府和市委的正确领导下，认真践行“三严三实”，弘扬“四种精神”和“三种特质”，以一天也不耽误的实干，用“双创”模式和动力，推动全市经济社会各项事业发展，为实现“十三五”良好开局，为海南科学发展、绿色崛起，扛起省会城市的担当，做出新的更大的贡献！
